# Supplementary material for: Overexpression of the vascular brassinosteroid receptor BRL3 confers drought resistance without penalizing plant growth
Source: Nat Commun. 2018 Nov 8;9:4680. doi: 10.1038/s41467-018-06861-3 (PMC6224425; doi:10.1038/s41467-018-06861-3)
Supplement: Supplementary file 1 — Supplementary Information [file 41467_2018_6861_MOESM1_ESM.pdf]

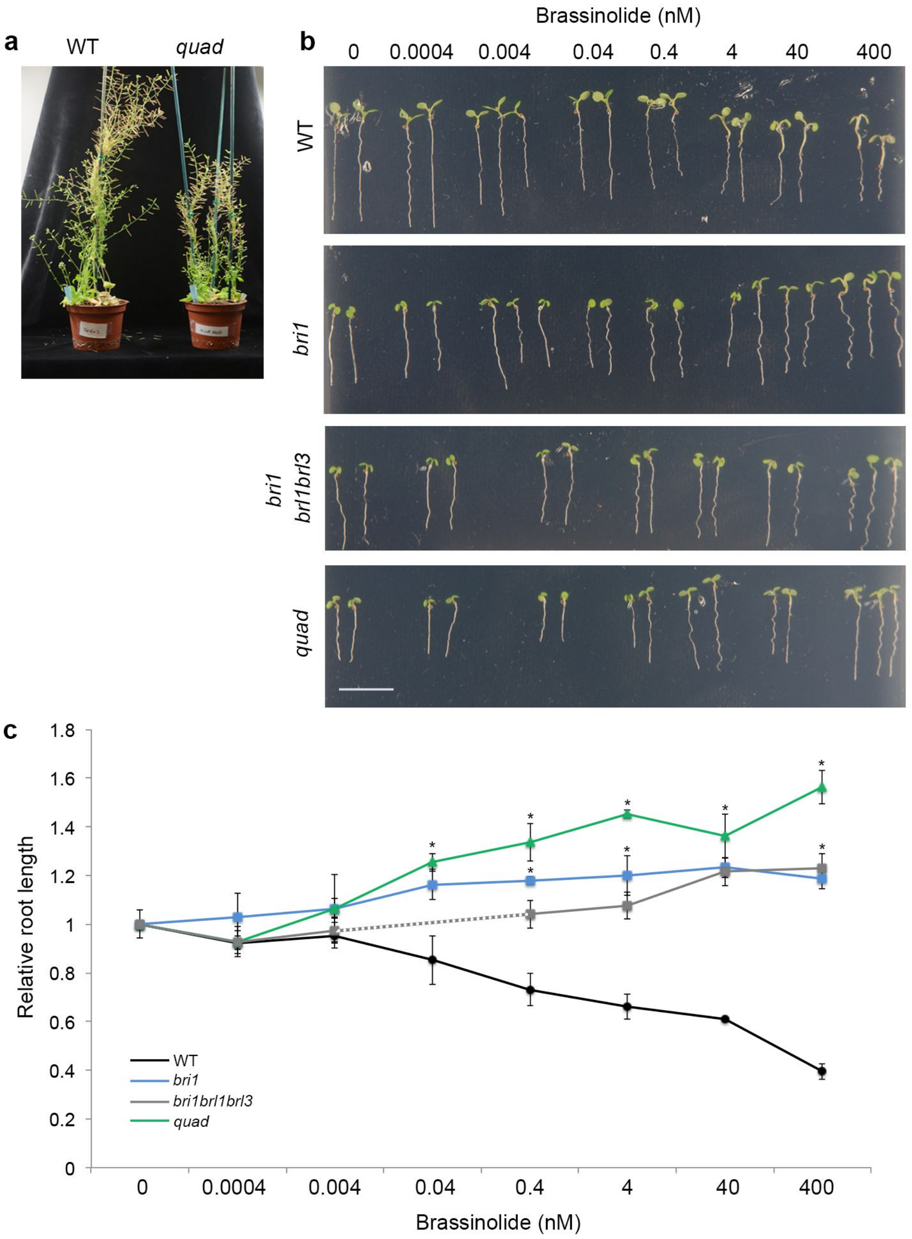

Supplementary Figure 1. BR loss-of-function *quad* mutant roots are insensitive to BRs.

(**a**) Five-week-old *quad* mutant plant phenotype. (**b**) Six-day-old roots of WT, *bri1*, *bri1brl1brl3*, and *quad* seedlings in a dose-response curve assay of 0.0004, 0.004, 0.04, 0.4, 4, 40, and 400 nM with brassinolide (BL). Scale bar: 1 cm.
(**c**) Relative root length of six-day-old WT, *bri1*, *bri1brl1brl3*, and *quad* seedlings within the BL dose-response curve. Root length in BL was normalized to control root length for each concentration and genotype. BL resistance is observed in *bri1*, *bri1brl1brl3* and *quad* roots. Results are the average of three independent biological replicates +/- S.E. (n=100). Asterisks denote statistical differences in a two-tailed t-test (p-value < 0.05).

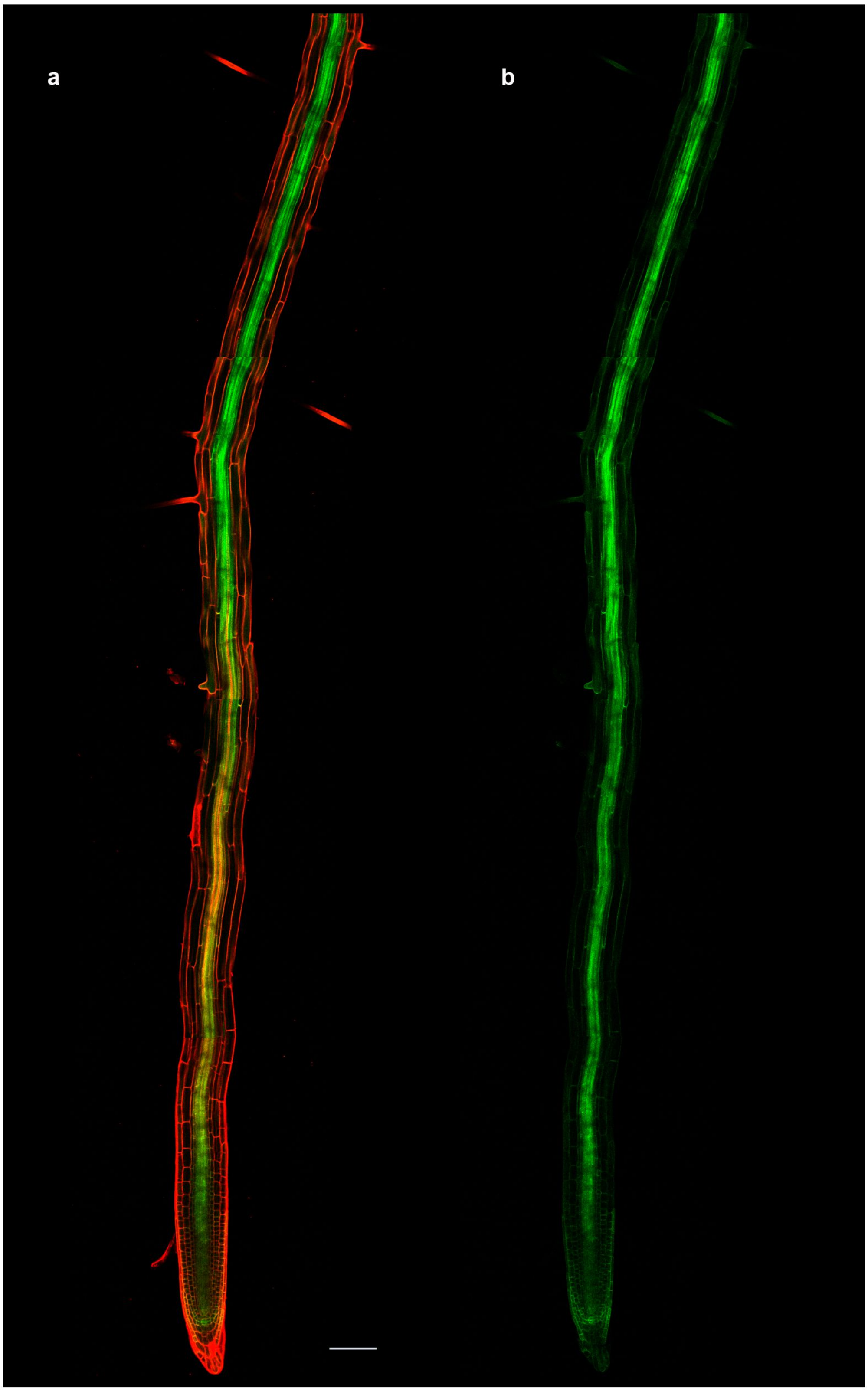

Supplementary Figure 2. Localization pattern of BRL3 protein in the *BRL3ox* roots.

(**a**) Longitudinal section of a *35S:BRL3-GFP* (*BRL3ox)* roots in confocal microscopy. The green channel depicts the GFP signal of the BRL3 receptor and the red channel depicts the PI staining of cell walls. Scale bar: 100µm.
(**b**) Same image shown in (**a**) but only the green channel showing the BRL3 localization pattern.

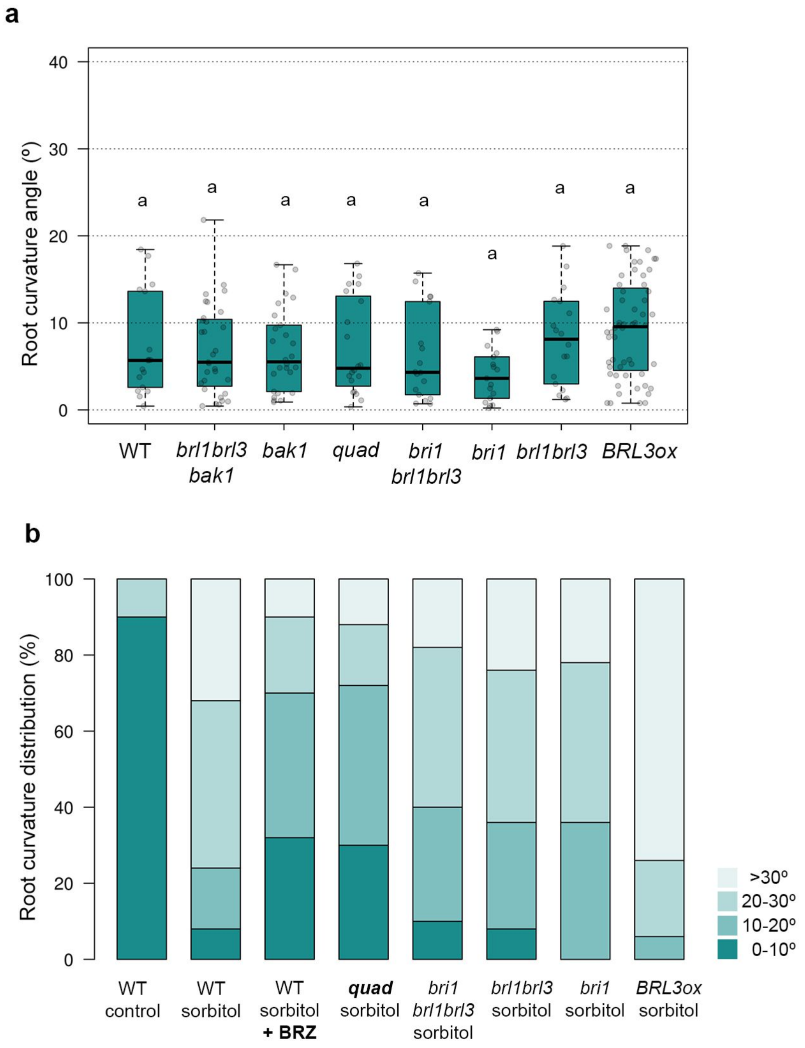

Supplementary Figure 3. Brassinazole reverts the WT root hydrotropic response to sorbitol.

(**a**) Curvature angles of roots when the bottom part of the media is replaced with control media (mock). Roots of all genotypes display a small curvature but show no differences compared with WT. Boxplot represent the median and interquartile range (IQR). Whiskers depict Q1-1.5*IQR and Q3+1.5*IQR. (n>35). Different letters represent significant differences (p-value<0.05) in a one-way ANOVA plus Tukey's HSD test. (**b**) Distribution of root curvature angles. Whereas 90% of the roots of WT plants in control conditions had curvatures lesser than 10º, only 8% of the roots of WT plants treated with 270 mM sorbitol had curvatures lesser than 10º (68% of the roots had curvatures between 0 and 30º). Exogenous application of brassinazole partially reverted the sorbitol induced response, with 33% of WT roots having curvatures below 10º and 90% of WT roots having curvatures below 30º. This distribution is similar to that of the insensitive *quad* mutants.

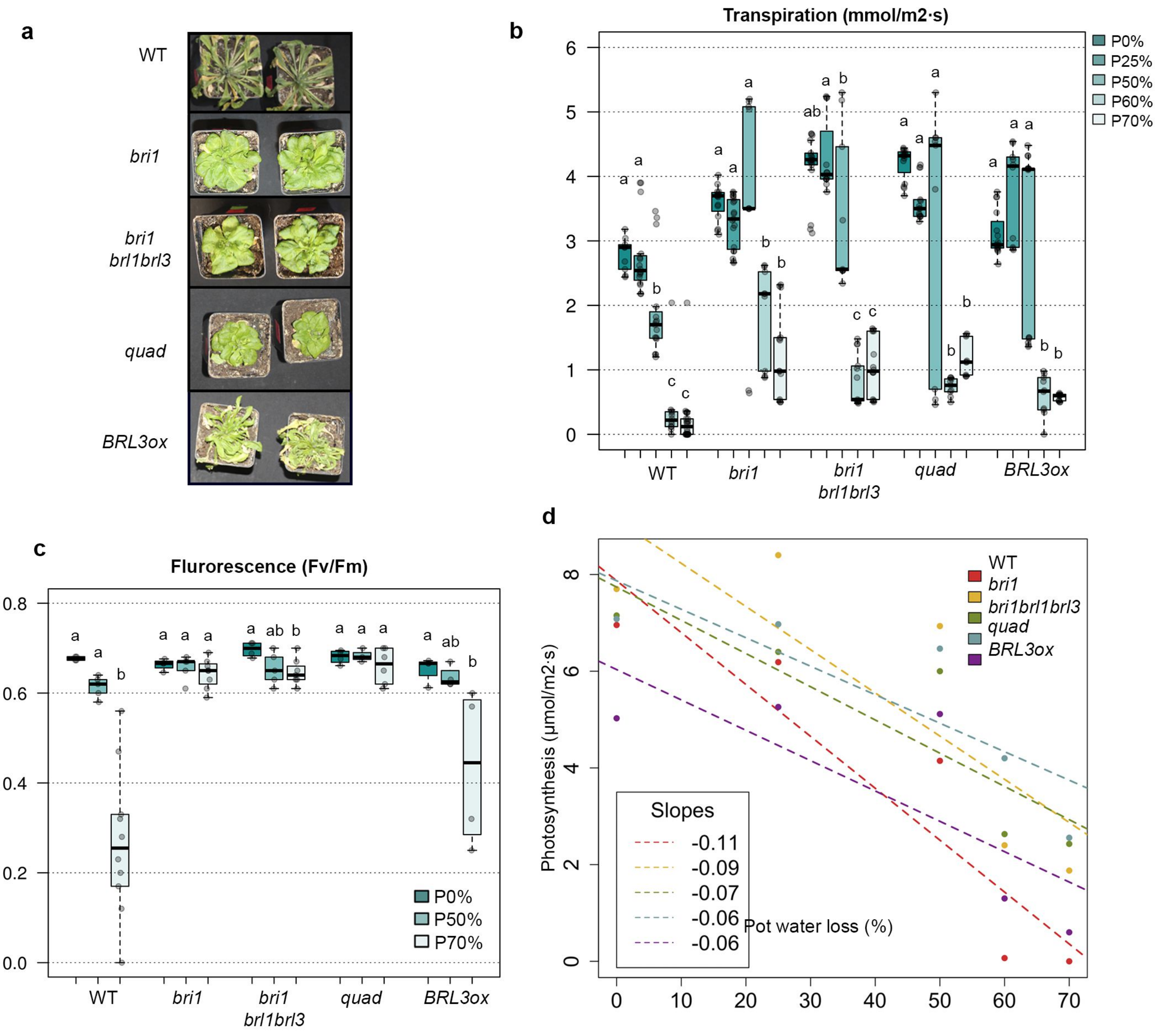

Supplementary Figure 4. Physiological parameters of mature plants under drought stress.

(**a**) Phenotypes of mature Arabidopsis plants subjected to drought. Rosettes were photographed when 70% soil water loss was reached (P70%) in each genotype. (**b**) Transpiration rates (mmol m-2 s-1) at different percentages of soil water loss. (**c**) Quantification of photosystem II (PSII) efficiency in mature rosettes at 0% (field capacity), 50% and 70% soil water loss. (**b, c**) Boxplot represent the median and interquartile range (IQR). Whiskers depict Q1-1.5*IQR and Q3+1.5*IQR. (n=6). Different letters depict significant differences (p-value<0.05) within each genotype in a one-way ANOVA plus a Tukey's HSD test. (**d**) Plot of photosynthesis efficiency versus soil water loss. Data was fitted to a lineal model and the slopes taken as indicators of drought-induced photosynthesis inhibition. Dots are the means for each genotype and dashed lines the fitted model.

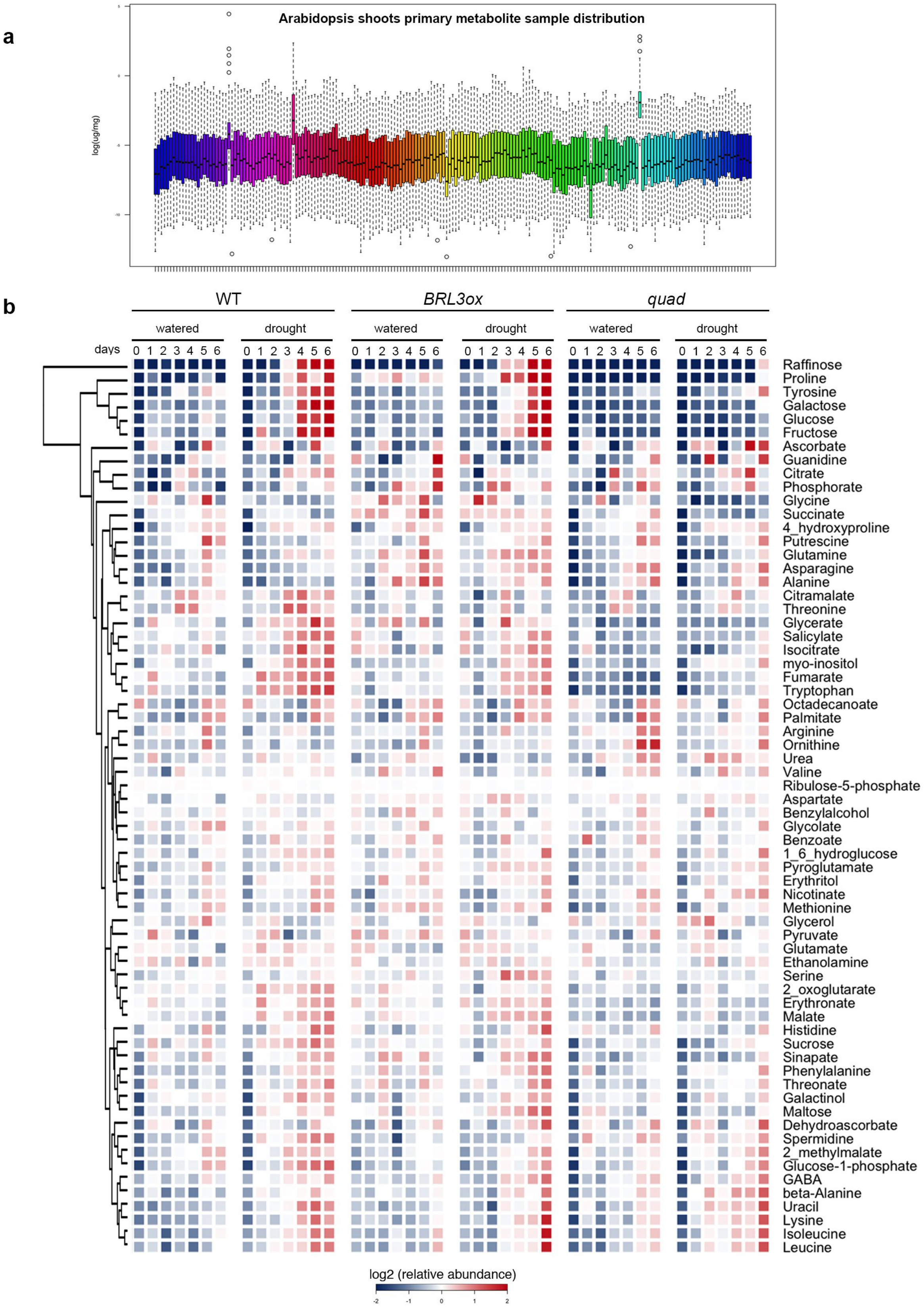

Supplementary Figure 5. Metabolite profiling of WT, *BRL3ox* and *quad* shoots under drought.

(**a**) Distribution of the complete sample set of metabolites in WT, *BRL3ox* and *quad* shoots within the drought time course. Data show a uniform distribution without normalization. Samples with median value outside the average interquartile range were not considered for further analysis. (**b**) Heat map depicts the metabolic fingerprint of WT, *BRL3ox* and *quad* shoots within the time course under control conditions and drought stress. While the relative metabolite levels are homogeneous in well-watered conditions, upon drought exposure, the metabolite patterns become deregulated. Color bar depicts row-scaled values for relative metabolite abundance.

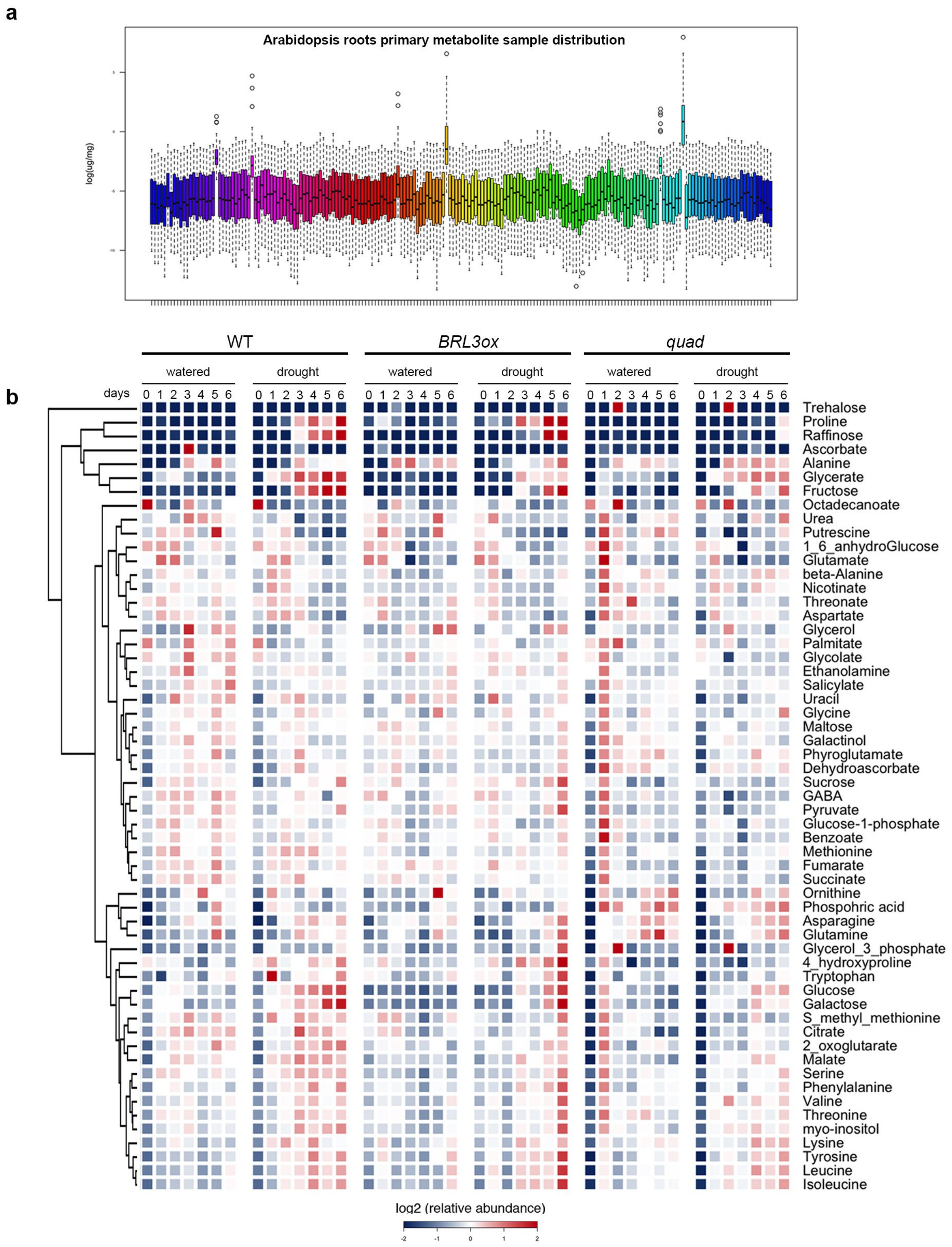

Supplementary Figure 6. Metabolite profiling of WT, *BRL3ox* and *quad* roots under drought.

(**a**) Distribution of the complete sample set of metabolites in WT, *BRL3ox* and *quad* roots within the drought time course. Data show a uniform distribution without normalization. Samples with median value outside the average interquartile range were not considered for further analysis. (**b**) Heat map depicts the metabolic fingerprint of WT, *BRL3ox* and *quad* roots within time course under control conditions and drought stress. While the relative metabolite levels are homogeneous in well-watered conditions, upon drought exposure, the metabolite patterns become deregulated. Color bar depicts row-scaled values for relative metabolite abundance.

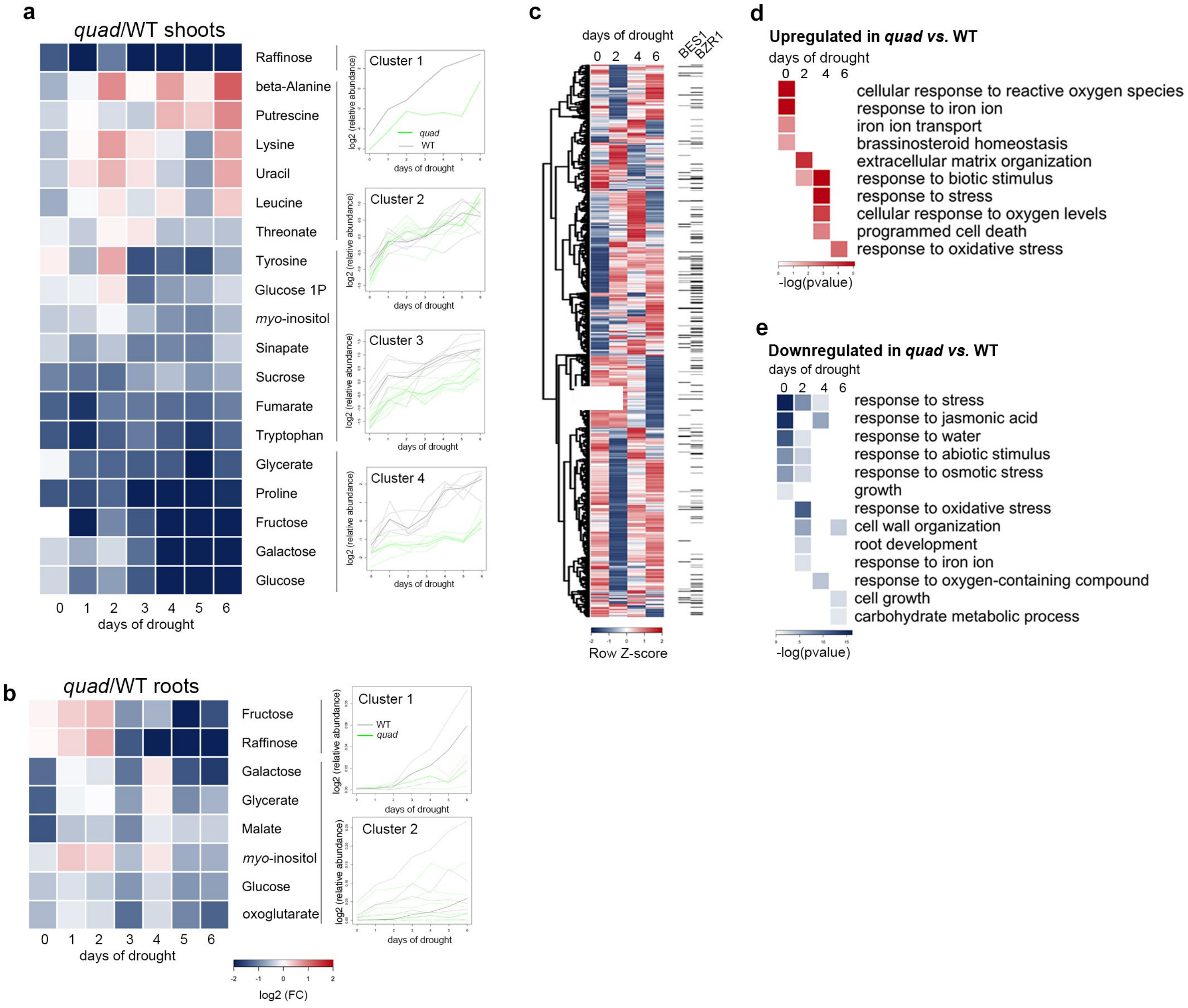

Supplementary Figure 7. Metabolomics and transcriptomics analyses of *quad* mutants under drought.

(**a**) Metabolites with differential dynamics between *quad* and WT shoots during the drought time course. (**b**) Metabolites with differential dynamics between *quad* and WT roots during the drought time course. (**a-b**) Heatmaps depict the log2 ratio of metabolite abundance in quad/WT. Clusters depict the dynamics of each metabolites. (**c**) Microarray analysis of mature quad rosettes during the drought time course. Heatmap represents row-normalized expression values. Black bars depict direct targets of BES1/BZR1. (**d**) Representative GO categories enriched among quad upregulated genes at 0, 2, 4, or 6 days of drought. (**e**) Representative GO categories enriched among quad downregulated genes at 0, 2, 4, or 6 days of drought. (**d-e**) Heatmaps represent the –log of corrected p-values for the enrichment (Benjamini-Hochberg).

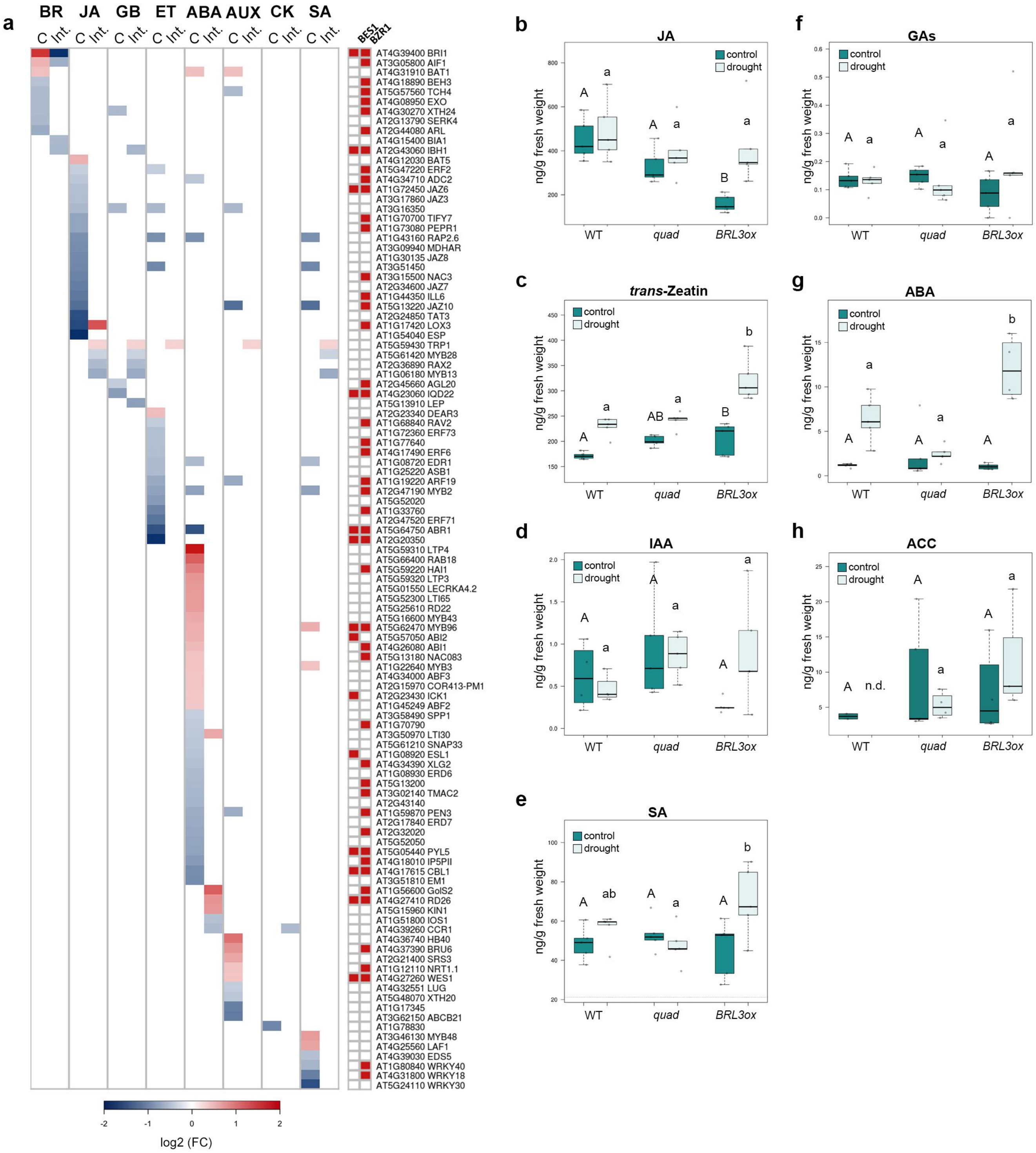

Supplementary Figure 8. Hormone profiling and the response of the deregulated genes to hormones in *BRL3ox* roots. (**a**) Genes within the "Response to hormone" (GO:0009725) term that are also annotated as responsive to brassinosteroids (BR), jasmonic acid (JA), gibberellins (GB), ethylene (ET), abscisic acid (ABA), auxin (AUX), citokinin (CK), or salicylic acid (SA). Colors in the heatmap represent the log2 fold change of *BRL3ox* roots versus WT in control (C) or the differential drought response (log2 (FC drought/CTRL in *BRL3ox*) – log2 (FC drought/CTRL in WT)) if the gene is affected by the interaction genotype*drought (Int.). Red color in the squared heatmaps to the right mean that the gene has previously been identified as a direct target of the BES1 or BZR1 transcription factors. (**b**) Levels of jasmonic acid (JA). (**c**) Levels of trans-zeatin, a type of cytokinin. (**d**) Levels of indole-3-acetic acid (IAA), the most common auxin. (**e**) Levels of salicylic acid (SA). (**f**) Total levels of gibberellic acid (GAs). (**g**) Levels of abscisic acid (ABA). (**h**) Levels of 1-aminocyclopropane-1-carboxylic acid (ACC), a precursor of ethylene biosynthesis. (**b-e**) Boxplot represent the median and interquartile range (IQR). Whiskers depict Q1-1.5*IQR and Q3+1.5*IQR and points experimental observations (n=5). Different letters depict significant differences (p-value<0.05) within each genotype in control (capital letters) or drought (lower-case letters) using a one-way ANOVA plus a Tukey's HSD post-hoc test.

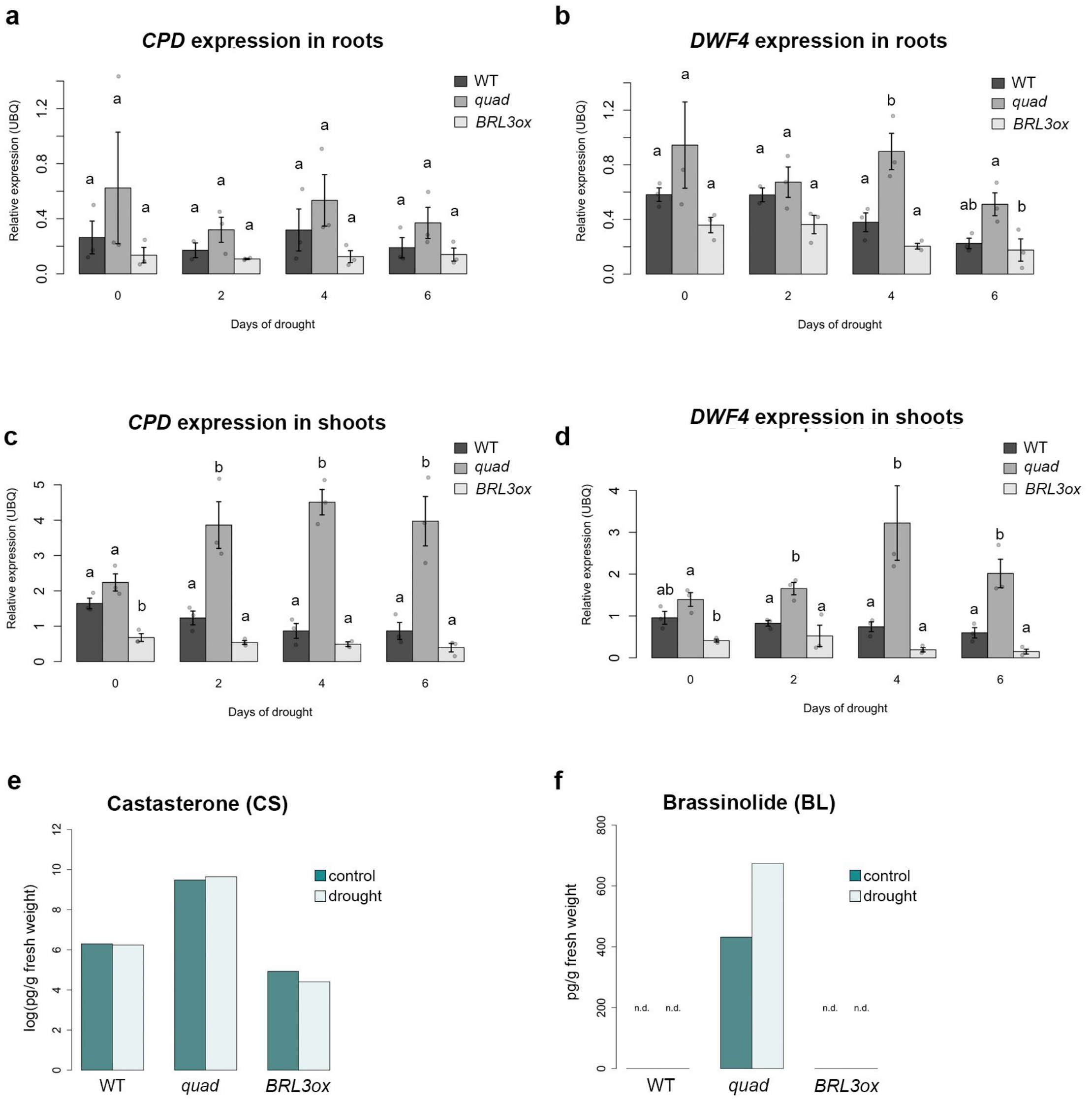

Supplementary Figure 9. BR pathway regulation in WT, *BRL3ox* and *quad* mutants under drought.

(**a**) *CPD* transcript levels in roots. (**b**) *DWF4* transcript levels in roots. (**c**) *CPD* transcript levels in shoots. (**d**) *DWF4* transcript levels in shoot. (**a-d**) Barplots repsent mean values and error bars +/- s.e.m. Points are experimental observations. Data from 3 independent biological replicates. Different letters mean significant differences (p-value<0.05) in one-way ANOVA test plus Tukey HSD poshoc test. (**e**) Castasterone (CS) levels. (**f**) Brassinolide (BL) levels. Data from a pool of >30 whole 28-days-old plants.

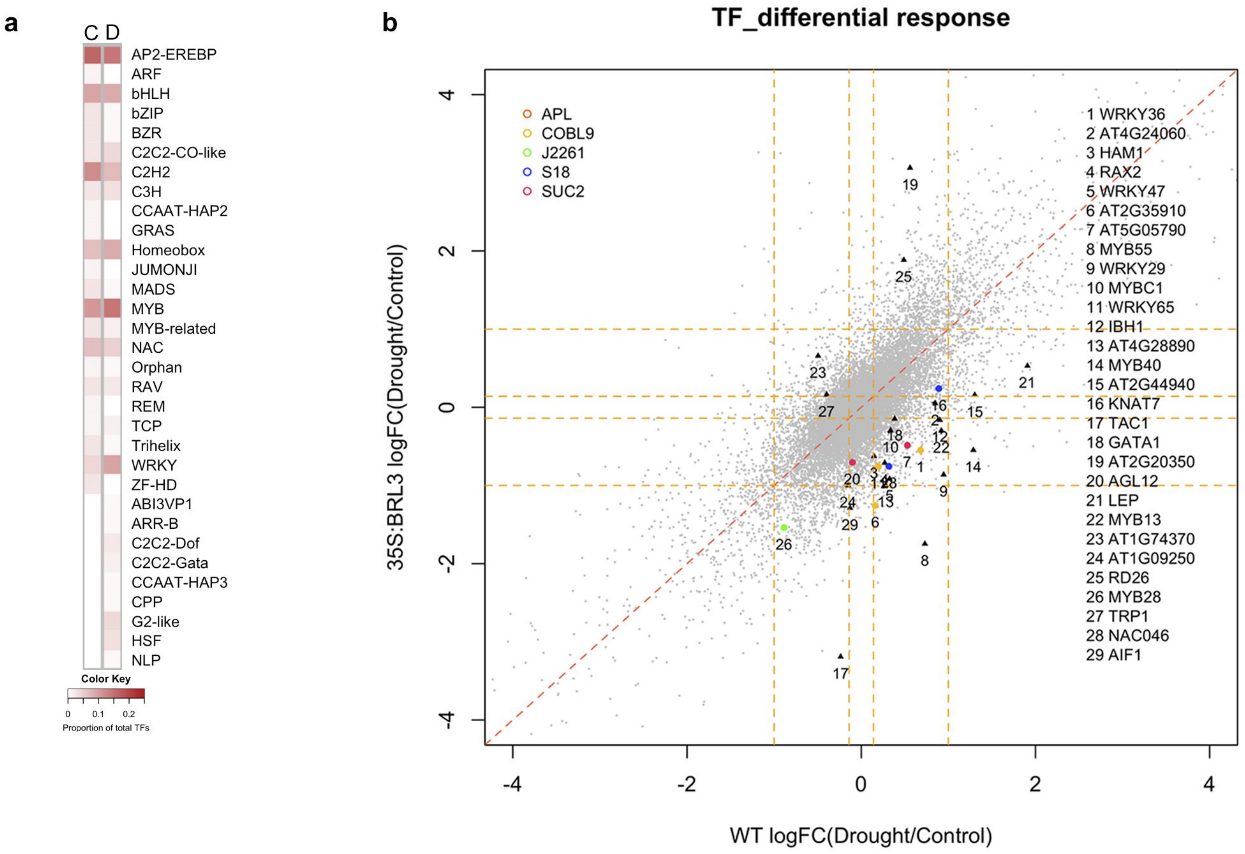

Supplementary Figure 10. Transcription factor analysis of the deregulated genes in *BRL3ox* roots upon drought.

(**a**) Families of deregulated transcription factors in control (left) and drought (right) conditions. Color key represents the proportion of the family in the total number of deregulated transcription factors. (**b**) Transcription factors with a differential response to drought in *BRL3ox* and WT roots (lineal model accounting for interaction). Root tissue-specific transcription factors are denoted in colors: APL, Phloem and phloem companion cells; COBL9, Root hair cells; J2261, Pericycle; S18, Maturing xylem; and SUC2, Phloem companions cells.

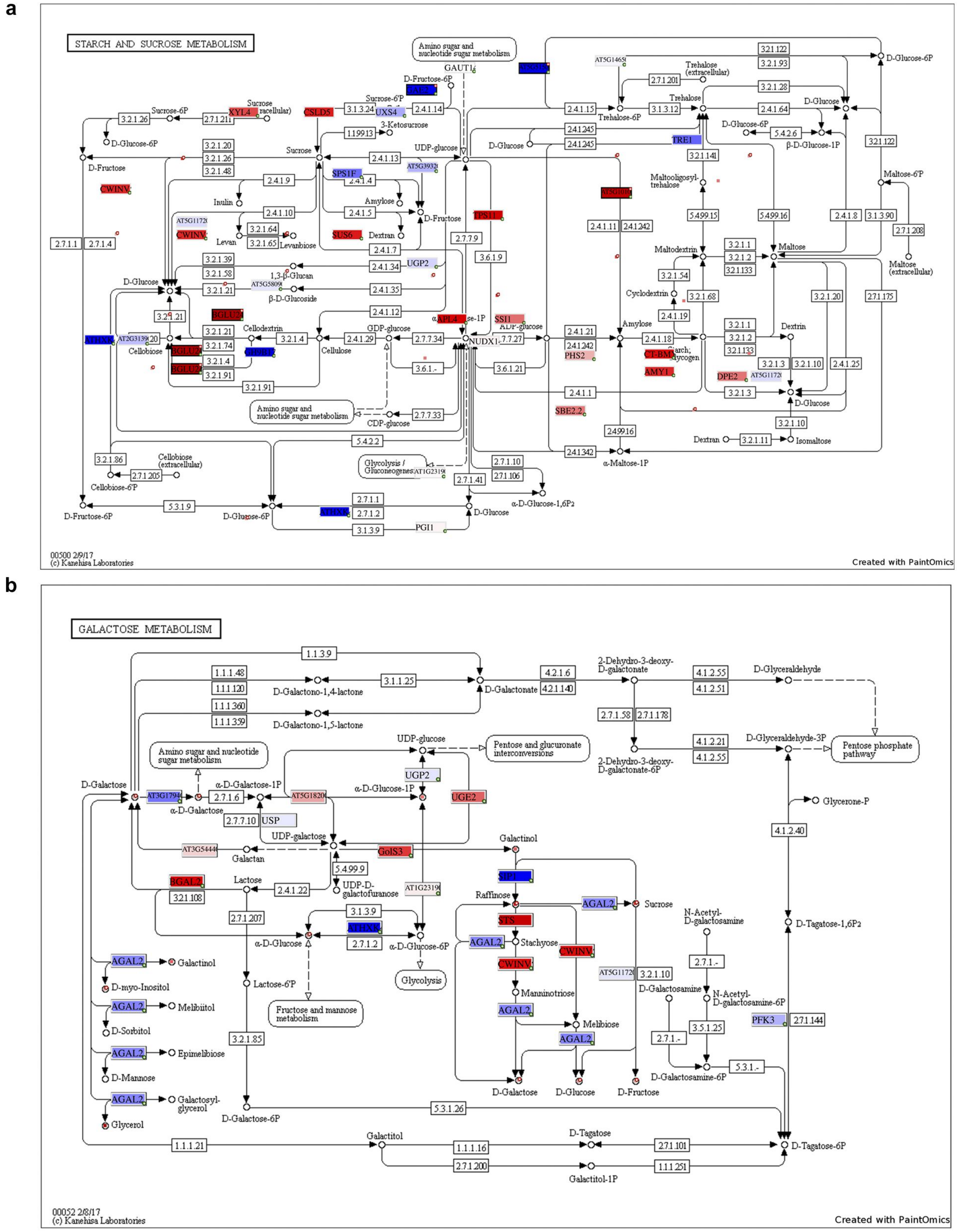

Supplementary Figure 11. The sucrose and galactose metabolic pathways are controlled by BRL3 under drought.

(**a**) Starch and sucrose metabolism (KEGG ath00500). Deregulated genes in *BRL3ox* roots are depicted in colored boxes between metabolites. (**b**) Galactose metabolism (KEGG ath00052). Deregulated genes in *BRL3ox* roots are depicted in colored boxes between metabolites. Metabolic pathways with the deregulated enzymes were generated with PaintOmics.

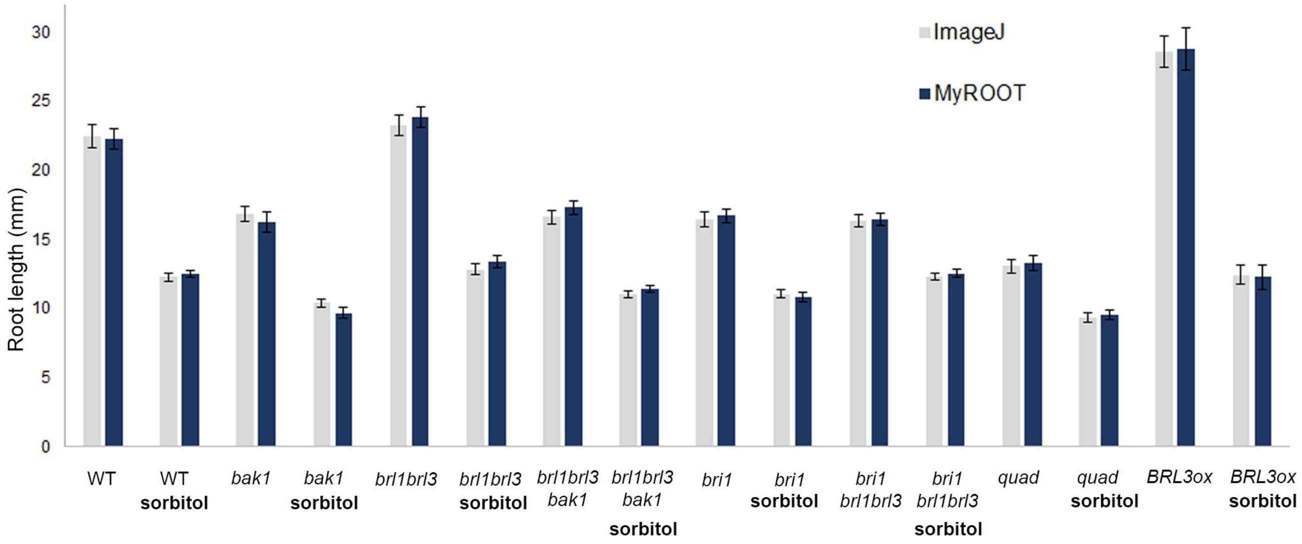

Supplementary Figure 12. Comparison of root length using ImageJ and MyROOT software.

Seven-day-old root length of WT, BR mutants and *BRL3ox* grown in control and sorbitol conditions. Manually measurements of root length were done with ImageJ and compared to data obtained by using the semi-automatic software MyROOT. No significant differences (t-test, p-value > 0.05) in the mean root lengths were observed between both methods. Bars represent means +/- S.E.

Supplementary Table 1: Raw data of the phenomic traits matrix shown in Figure 2d

		WT	*bak1*	*bri1*	*brl1brl3*	*brl1brl3bak1*	*bri1brl1brl3*	*quad*	*BRL3ox*
**Plant survival**	% surviving plants after drought	0,160	0,476	0,646	0,182	0,195	0,754	0,766	0,342
**Hydrotropic sensitivity**	Mean angle (º)	24,382	15,464	19,585	23,223	10,448	18,435	15,626	29,906
**Sorbitol resistance**	Ration root length sorbitol/control	0,611	0,656	0,733	0,663	0,679	0,724	0,730	0,633
**PCD sensitivity**	Ratio PI-stained area sorbitol/control	4,136	3,766	2,095	4,723	3,394	2,695	2,220	6,063
**Root length**	cm	2,544	1,741	2,070	2,567	1,840	1,800	1,530	2,951

Supplementary Table 2. Primers used for genotyping

**primer**	**sequence**	**mutant**
bri2.11	GTGAGAAACGAAGGTGGAACAGACTGCAG	*brl1-1*
bri2.12	CTTCTTGGCATGGATACGGGAGGTAATGG	*brl1-1*
JMLB	GGCAATCAGCTGTTGCCCGTCTCACTGGTG	*brl1-1*
bri4.2	TTTAGGGTGAGCATGAGATCTCGTGGGCCG	*brl3-1*
bri4.3	GAAATCCCTGTAGGAATCGGAAAGCTTGAG	*brl3-1*
JMRB	GCTCATGATCAGATTGTCGTTTCCCGCCTT	*brl3-1*
bak1-3 F	GCACTGAAAAACAGTTTAGC	*bak1-3*
bak1-3 R	GATGCAGGAAGGGGAGTCAACTTGGTG	*bak1-3*
T-DNA R	GCGTGGACCGCTTGCTGCAACT	*bak1-3*
bri1-301 F	GGAAACCATTGGGAAGATCA	*bri1-301 **
bri1-301 R	GCTGTTTCACCCATCCAA	*bri1-301 **

*PCR product was digested by *DpnII* enzyme (R0543, New England Biolabs)

Supplementary Table 3. Primers used for RT- qPCR

primer	sequence	transcript
CPD F	AGAGCGGTTCATTTAGACCCA	*CPD*
CPD R	TACCGAGTTGCTCTGCCATC	*CPD*
DWF4 F	CTCAGCCGTGGAACATTTGG	*DWF4*
DWF4 R	AACAACGGAGCGTCATCCTC	*DWF4*
