# Supplementary material for: Overexpression of the vascular brassinosteroid receptor BRL3 confers drought resistance without penalizing plant growth
Source: Nat Commun. 2018 Nov 8;9:4680. doi: 10.1038/s41467-018-06861-3 (PMC6224425; doi:10.1038/s41467-018-06861-3)
Supplement: Supplementary file 2 — Description of Additional Supplementary Files [file 41467_2018_6861_MOESM2_ESM.pdf]

Description of Additional Supplementary Files:

Supplementary Data File 1: Metabolites identified in the shoot. Data normalized by sample fresh weight and internal standard (Ribitol)

Supplementary Data File 2: Metabolites identified in the root. Data normalized by sample fresh weight and internal standard (Ribitol)

Supplementary Data File 3: Deregulated genes of RNAseq at basal conditions BRL3ox vs. WT

Supplementary Data File 4: Deregulated genes of RNAseq after 5 days of drought BRL3ox vs. WT

Supplementary Data File 5: GO enrichment for Biological Process ontology at basal conditions, upregulated genes in BRL3ox vs. WT

Supplementary Data File 6: GO enrichment for Biological Process ontology at basal conditions, down regulated genes in BRL3ox vs. WT

Supplementary Data File 7: GO enrichment for Biological Process ontology upon 5 days of drought, upregulated genes in BRL3ox vs. WT

Supplementary Data File 8: GO enrichment for Biological Process ontology upon 5 days of drought, down regulared in BRL3ox vs. WT

Supplementary Data File 9: Interaction-affected genes *logFC= log(FC WT drought/WT control)-log(FC BRL3ox drought/BRL3ox control)

Supplementary Data File 10: GO enrichment for Biological Process ontology on interaction-affected genes, being more upregulated upon drought in BRL3ox than in WT

Supplementary Data File 11: GO enrichment for Biological Process ontology on interaction-affected genes, being more upregulated upon drought in WT than in BRL3ox

Supplementary Data File 12: Deregulated genes in quad vs. Col-0 WT at basal control conditions

Supplementary Data File 13: Deregulated genes in quad vs. Col-0 WT at 2 days of drought

Supplementary Data File 14: Deregulated genes in quad vs. Col-0 WT at 4 days of drought

Supplementary Data File 15: Deregulated genes in quad vs. Col-0 WT at 6 days of drought

Supplementary Data File 16: Paintomics results of transcriptomics-metabolomics integration of BRL3ox vs. Col0 WT at basal control conditions

Supplementary Data File 17: Paintomics results of transcriptomics-metabolomics integration of BRL3ox vs. Col0 WT under drought
